# Biology Open 2024 – a year in review

**DOI:** 10.1242/bio.062121

**Published:** 2025-07-02

**Authors:** Daniel A. Gorelick

**Affiliations:** Editor-in-Chief, Biology Open

How do scientists choose where to submit a manuscript for peer review and publication? There are many factors, but I think that the author experience is important. At the start of my appointment as Editor-in-Chief in 2023, I hypothesized that if the author experience at Biology Open (BiO) is substantially better than that at other journals, then authors will be more likely to submit to BiO. How are we improving the author experience? BiO offers rapid peer review, rigorous peer review and transparency. In 2023, we published transparent criteria that we use to make editorial decisions on manuscripts (Gorelick, 2023). In this Editorial, I will review our performance for the year 2024.

## What did we publish?

**Fig. 1.**
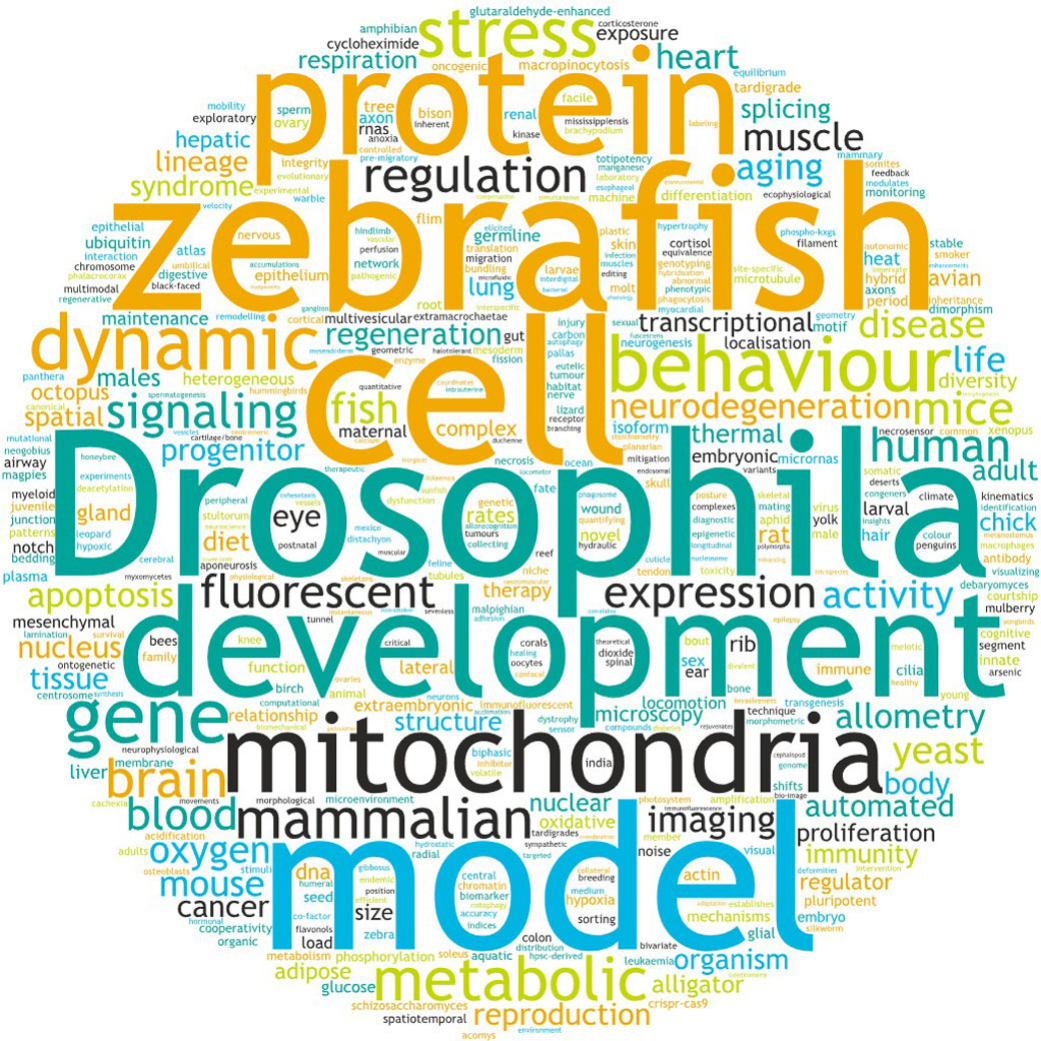
Word cloud based on key words of articles published in BiO during 2024.

BiO publishes peer-reviewed original research in all areas of the biological and biomedical sciences. Fig. 1 shows a representative set of topics and keywords from all research articles published in 2024. *Drosophila*, zebrafish and development were especially popular – no surprise considering that our publisher, The Company of Biologists, is known for publishing outstanding papers in developmental biology, using model systems like *Drosophila* and zebrafish.

## How good was the author experience?

Before diving into the data, a caveat. In 2024, BiO undertook two major changes that made it more challenging to assess author experience using consistent metrics.

First, midway through 2024, we transitioned from our previous workflow management system (BenchPress) to a new one (Editorial Manager). The two systems do not align at all. They track and present data differently, making direct comparisons difficult. Despite these challenges, the BiO staff went above and beyond. They manually reviewed and reconciled data from both systems to ensure that this report is as accurate and representative as possible.

Second, in July 2024, we launched an experiment to explore the feasibility of delivering fast, high-quality peer review, an initiative we called Fast & Fair peer review (Gorelick and Clark, 2025). Under this model, all manuscripts assigned to two of our academic editors were processed through an accelerated workflow: authors received first decisions with reviews within 7 working days of submission. All other submissions continued through our conventional peer review workflow.

The data presented below include all manuscripts, regardless of whether they were managed in BenchPress or Editorial Manager or whether they underwent Fast & Fair or conventional peer review. Only 7% of submitted manuscripts in 2024 followed the Fast & Fair path, so the overall statistics are not significantly impacted by this pilot.

Our 2024 publication data are summarized in Table 1. When compared to those from 2023, the figures are broadly consistent. Our overall acceptance rate remains at approximately 30%. Notably, we saw an improvement in the median time from submission to editorial rejection (rejection without peer review), which dropped from 7 days in 2023 to 3 days in 2024. However, there was a modest increase in the median time from submission to decision following peer review, which rose from 33 to 39 days.

**Table 1.** BiO publication statistics for Research Articles and Methods & Techniques articles for 2023 and 2024

Research manuscript statistics (excludes reviews)	2023	2024
Submissions (total, including transfers)	308	365
Published articles	100	129
Overall acceptance rate	35%	33%
Editorial rejections	142	136
Submissions sent for peer review	144	208
Direct submissions	174	241
Transferred submissions	134	124
Transfers without reviews	81	99
Transfers with reviews	53	25
Transferred articles accepted without additional peer review	22	17
Transferred articles accepted (total)	51	58
Peer review statistics (days)		
Submission to final decision (mean±s.d.)	70±54	77±60
Submission to final decision (median)	59	65
Submission to editorial rejection (without peer review) (mean)	10±11	10±23
Submission to editorial rejection (without peer review) (median)	7	3
Submission to first decision following peer review (mean)	34±26	46±41
Submission to first decision following peer review (median)	33	39

## How would I assess our performance in 2024?

Based on our overall journal timings, we have not seen a significant improvement at the journal level. As I mentioned in last year's Editorial (Gorelick, 2023), peer review should not take a month, and editorial rejections should not take a week. This is why we are testing the feasibility of rapid and high-quality peer review. Through our Fast & Fair peer review pilot, we pre-contracted and paid reviewers, enabling us to deliver decisions with high-quality reviews within 7 working days of submission.

Although the number of manuscripts processed through Fast & Fair peer review in 2024 was too small to influence our overall journal metrics, the early results are compelling (Gorelick and Clark, 2025). Across all 20 manuscripts included in the pilot, the average time from submission to first decision with peer reviews was 4.6 business days, ranging from 2 to 7 days. That is approximately ten times faster than our standard peer review timeline.

Encouraged by these results, we began expanding Fast & Fair peer review to other areas of the journal in 2025. We are now in Phase II of the experiment, focusing on testing the scalability of this approach. Our goal is ambitious but clear: we want every manuscript submitted to BiO to be considered through the Fast & Fair peer review workflow. We will be sharing preliminary results of Phase II of the experiment soon, so stay tuned.

None of this would be possible without the dedication and hard work of our staff, academic editors and peer reviewers, who have made 2024 a special year for the journal. Special thanks to all our peer reviewers, who are listed in the supplementary information. Thank you all for your dedication to BiO and The Company of Biologists.

Since launching Fast & Fair in July 2024, months before the US presidential election, the research climate in the United States has grown more challenging. The government has rescinded existing grants and signalled deep cuts to future funding, while recent executive orders and proposed legislation threaten to curtail opportunities for scientists, especially those from historically under-represented groups, and to deter international scholars from studying or working in the United States. Against challenges of this scale, reforming peer review can feel like a luxury. Yet, as my grandmother would remind me, “Der mentsh trakht un got lakht” – people plan and the universe laughs. We carry on because peer review needs fixing, and we want a proven, scalable solution for researchers everywhere, whatever becomes of the US research landscape.

## Supplementary Material

10.1242/biolopen.062121_sup1 Supplementary information
