# Supplementary material for: Biology Open 2024 – a year in review
Source: Biol Open. 2025 Jul 2;14(6):bio062121. doi: 10.1242/bio.062121 (PMC12264732; doi:10.1242/bio.062121)
Supplement: Supplementary information [file biolopen-14-062121-s1.pdf]

Reviewers for Biology Open 2024

Mohammad Abbas, Aligarh Muslim University, India

Fernando Abdulkader, University of São Paulo, Brazil

Elizabeth Ables, East Carolina University, USA

Masood Abu-Halima, Saarland University, Germany

Joaquín Acedo, University of Basel, Switzerland

Natsumi Ageta-Ishihara, Toho University, Japan

Francesco Agostini, University of Padua, Italy

Mohammad Vahid Ahmadianpour, National Institute of Genetic Engineering and Biotechnology, Islamic Republic of Iran

Henriette Aksnes, University of Bergen, Norway

Abdullah Al-Sadi, Sultan Qaboos University, Oman

João Miguel Alves-Nunes, São Paulo State University, Brazil

Alwin Prem Anand, University of Tübingen, Germany

Cynthia Andoniadou, King's College London, UK

Mariana Angoa-Perez, Wayne State University, USA

FNU Anupriya, Wake Forest University, USA

Luca Argiro, Max Planck Institute for Plant Breeding Research, Germany

Alissa Armstrong, University of South Carolina, USA

Sagar Arya, Khalifa University, United Arab Emirates

Naghmana Ashraf, Baylor College of Medicine, USA

Birol Baki, Sinop University, Turkey

Vasco Barreto, NOVA School of Science and Technology, Portugal

Steve Bassnett, Washington University in St Louis, USA

Elizabeth Bastiaans, State University of New York at Oneonta, USA

Evalyn Beall, University of Minnesota, USA

Camilla Bean, University of Udine, Italy

Rachel Bear, Emory University, USA

Christian Behrends, Ludwig Maximilian University of Munich, Germany

Tobias Beigl, Robert Bosch Center for Tumor Diseases, Germany

Biology Open • Supplementary information

Arica Beisaw, Heidelberg University, Germany

John Belcher, University of Minnesota, USA

Sandra Bensmihen, University of Toulouse, France

Dan Bergstralh, University of Missouri Columbia Health Care, USA

Stéphane Betoulle, University of Reims Champagne-Ardenne, France

Martin Beye, Heinrich Heine University Düsseldorf, Germany

Nicolas Bidere, Inserm Research Institute, France

Marco Bisaglia, University of Padua, Italy

Sean Blamires, University of New South Wales, Australia

Michelle Bland, University of Virginia, USA

Cecilia Bucci, University of Salento, Italy

Alex Callen, The University of Newcastle, Australia, Australia

Barbara Cannon, Stockholm University, Sweden

Guy Carpenter, King's College London, UK

Tamara Caspary, Emory University, USA

Flore Castellan, NYU Langone Health, USA

Maureen Cetera, University of Minnesota, USA

Joy Chakraborty, Indian Institute of Chemical Biology CSIR, India

Kishore Bhallagundla, University of Nebraska Medical Centre, USA

Preethi Chandrasekaran, University of Texas Southwestern Medical Center, USA

James Charles, University of Liverpool, UK

Garima Chauhan, Indian Institute of Technology Kanpur, India

Claire Chazaud, Clermont Auvergne University, France

Xiaobing Chen, National Institute of Neurological Disorders and Stroke (NIH), USA

Xie Jie Chen, State University of New York Upstate Medical University, USA

Xiumin Chen, Chinese Academy of Agricultural Sciences, China

Yao Chen, Xianning Vocational Technical College, China

Rui Cheng, Wake Forest University, USA

Leon Chew, University of British Colombia, Canada

Valentina Cianfanelli, Roma Tre University, Italy

Kenneth Clark, West Chester University, USA

Biology Open • Supplementary information

Michelle Collins, University of Saskatchewan, Canada

David Costantini, Tuscia University, Italy

Naomi Courtemanche, University of Minnesota, USA

Rachel Cox, Uniformed Services University of the Health Sciences, USA

James Crall, University of Wisconsin-Madison, USA

James Cray, Ohio State College of Medicine, USA

Ondi Crino, Flinders University, Australia

Dane Crossley II, University of North Texas, USA

Ales Cvekl, Albert Einstein College of Medicine, USA

Ira Daar, National Cancer Institute (NCI), USA

Richard Dahl, Indiana University School of Medicine, USA

Nico Dantuma, Karolinska Institute, Sweden

Charles Darveau, University of Ottawa, Canada

Subham Dasgupta, Clemson University, USA

Sayantan Datta, Emory School of Medicine, USA

Chiara De Gregorio, University of Turin Italy

Joaquín de Navascués, University of Essex, UK

Jan de Vries, University of Göttingen, Germany

Mark DeCoster, Louisiana Tech University, USA

Nina Dehnhard, Norwegian Institute for Nature Research, Norway

Javier delBarco-Trillo, Illinois State University, USA

Rebecca Delventhal, Lake Forest College, USA

Nicole DesJardins, University of Wisconsin-Madison, USA

Valentina Di Santo, Stockholm University, Sweden

Robert Dilley, Massachusetts General Hospital, USA

Eimear Dolan, University of São Paulo, Brazil

Yaprak Dönmez Çakıl, Maltepe University, Turkey

Callum Donohue, Murdoch University, Australia

Laurent Duchatelet, Catholic University of Louvain, Belgium

Maxime Durand, University of Helsinki, Finland

Atanu Duttaroy, Howard University, USA

Klaus Ebnet, University of Münster, Germany

Martin Egan, University of Arkansas, USA

Olaf Ellers, Bowdoin College, USA

Kyle Elliott, McGill University, Canada

Rosalee Elting, University of Montana, USA

Ludovic Enkler, University of Strasbourg, France

Saskia Erttmann, Umeå University, Sweden

John Ewer, University of Valparaíso, Chile

Andreas Fahlman, Oceanogràfic de València, Spain

Peter Flynn, Harvard University, USA

Morten Frost Nielsen, Odense University Hospital, Denmark

B. M. Gadella, Utrecht University, Netherlands

Gina Galli, The University of Manchester, UK

Brigitte Galliot, University of Geneva, Switzerland

Sayantika Ghosh, University of Cambridge, UK

Edward Giniger, National Institutes of Health (NIH), USA

Fernando Gomes, University of São Paulo, Brazil

Kelly Gomez-Campo, Penn State Eberly College of Science, USA

Agustina Gómez-Laich, National Scientific and Technical Research Council (CONICET), Argentina

Victor Gonzalez, The University of Kansas, USA

Juan Manuel Gonzalez-Rosa, Boston College, USA

Jeffrey Good, University of Auckland, New Zealand

Mubeen Goolam, University of Cape Town, South Africa

Daniel Gorelick, Baylor College of Medicine, USA

Cara Gottardi, Northwestern University, USA

Kathleen Gould, Vanderbilt University, USA

Iva Greenwald, Columbia University, USA

H. Leighton Grimes, Cincinnati Children's Hospital Medical Center, USA

Paul Grimshaw, Hamad Bin Khalifa University, Qatar

Adam Grodek, University at Buffalo, USA

Jianjun Gu, The Second Affiliated Hospital of Nantong University, China

Jordi Guiu, Bellvitge Biomedical Research Institute (IDIBELL), Spain

Saumya Gupta, University of Washington, USA

Madalene Halley, University of Minnesota, USA

Shelley Halpain, University of California, San Diego, USA

Peter Hansen, University of Florida, USA

Emily Hardison, University of Pittsburgh, USA

Tony Harris, University of Toronto, Canada

Klaus Hartfelder, University of São Paulo, Brazil

Rui He, Beijing University of Chinese Medicine, China

Tyson Hedrick, University of North Carolina at Chapel Hill, USA

Greg Hermann, Lewis & Clark College, USA

Marcelo Hermes-Lima, University of Brasilia, Brazil

Anthony Herrel, National Museum of Natural History, France

Johannes Herrmann, University of Kaiserslautern, Germany

Coen Hird, The University of Queensland, Australia

Thomas Holstein, Heidelberg University, Germany

Sachiko Homma-Takayama, Boston University School of Medicine, USA

Martin Hora, Charles University in Prague, Czech Republic

Hadley Horch, Bowdoin College, USA

Tai-I Hsu, National Cheng Kung University, Taiwan

Anna Hughes, University of Essex, UK

Hye Jin Hwang, Uniformed Services University, USA

Naoko Isomura, National Institute of Technology, Okinawa College, Japan

William Ja, Scripps Research, USA

Cathy Jackson, Institut Jacques Monod, France

Raphaella Jackson, Imperial College London, UK

Logan James, McGill University, Canada

Jeremy Jamieson, University of Rochester, USA

Silvia Jansen, Washington University in St Louis, USA

Jane Jardine, INSERM Research Institute, France

Mohammed Jasim, University of Anbar, Iraq

Annie Jessop, Murdoch University, Australia

Lin Jiao, Sichuan University, China

Granton Jindal, University of California, San Diego, USA

Joaquina Delás, Francis Crick Institute, UK

Henry John-Alder, Rutgers University, USA

Keith Johnson, University of Nebraska Medical Center, USA

Darryl Jones, Griffith University, Australia

Nathalie Jurisch-Yaksi, Norwegian University of Science and Technology, Norway

Fredrik Jutfelt, Norwegian University of Science and Technology, Norway

Younes Kamali, Ferdowsi University of Mashhad, Islamic Republic of Iran

Brittany Katz, University of California, Los Angeles, USA

Risa Kawaguchi, Kyoto University, Japan

Markku Keinänen, University of Eastern Finland, Finland

Yasmine Kemkem, King's College London, UK

Pranav Khandelwal, Virginia Polytechnic Institute and State University, USA

Shaun Killen, University of Glasgow, UK

Donghwan Kim, Korea Food Research Institute, Republic of Korea

Michael Kimmich, State University of New York Upstate Medical University, USA

Janine Kirstein, Leibniz Institute on Aging, Germany

Roger Kissane, University of Liverpool, UK

Ine Kjosås, University of Bergen, Norway

Christoph Kleineidam, University of Konstanz, Germany

Hyongjong Koh, Dong-A University College of Medicine, Republic of Korea

Dennis Kolosov, California State University San Marcos, USA

Konstantinos Kormas, University of Thessaly, Greece

Vladimír Košťál, Biology Centre of the Czech Academy of Sciences, Czech Republic

Patricia Kramer, University of Washington, USA

Mira Krendel, State University of New York Upstate Medical University, USA

Emma Kromann, King's College London, UK

Urszula Krzych, Military Health System, USA

Abdulsamed Kükürt, Kafkas University, Turkey

Adam Kwiatkowski, University of Pittsburgh, USA

Hye-Joo Kwon, The University of Utah, Asia Campus, Republic of Korea

Raj Ladher, National Centre for Biological Sciences (NCBS), India

Benjamin Le Vely, University of Nantes, France

Lyndsay Leach, The University of Texas at Austin, USA

Choon Lee, University of Malaya, Malaysia

Tina Lee, Tan Tock Seng Hospital, Singapore

Youngsoo Lee, Ajou University School of Medicine, Republic of Korea

Véronique Lefebvre, Children's Hospital of Philadelphia, USA

Philipp Lehmann, Stockholm University, Sweden

Nicholas Leigh, Lund University, Sweden

Félix Leiva, Radboud University, Netherlands

Holger Lerche, University of Tübingen, Germany

Kornkamon Lertsuwan, Mahidol University, Thailand

Michael Levin, Tufts University, USA

Lindsay Lewellyn, Butler University, USA

Kefei Li, Cornell University, USA

Zhenmin Li, Shandong University, China

Iris Lindberg, University of Maryland School of Medicine, USA

Lin Liu, Nankai University, China

Yi-Shi Liu, Jiangnan University, China

Hin Ching Lo, AbbVie Inc., USA

Jeremy Logue, Albany Medical College, USA

Ben Lovely, University of Louisville, USA

Marjorie Lundgren, Lancaster University, UK

Yiping Luo, Southwest University, China

Rendani Luthada-Raswiswi, University of KwaZulu-Natal, South Africa

Huub Maas, Free University of Amsterdam, Netherlands

Laura Machesky, University of Cambridge, UK

Chris Maher, Washington University School of Medicine, USA

Hans Malte, Aarhus University, Denmark

Zoe Mann, King's College London, UK

Miguel Manzanares, Severo Ochoa Molecular Biology Center, Spain

Hendrik Marks, Radboud University, Netherlands

James Marrs, Indiana University, USA

Francis Martin, Blackpool Victoria Hospital, UK

Graham Martin, University of Birmingham, UK

Jeff Martin, South Dakota State University, USA

Juan Pedro Martinez-Barbera, University College London, UK

L. Miguel Martins, University of Cambridge, UK

Elvira Mass, University of Bonn, Germany

Karl Matter, University College London, UK

Mark McCormick, The University of New Mexico, USA

Richard Meitern, University of Tartu, Estonia

Bruno Melo, American Museum of Natural History, USA

Grace Melone, University of Wisconsin-Madison, USA

Christa Merzdorf, Montana State University, USA

Marco Milán, Institute for Research in Biomedicine, Spain

Nobuaki Mizumoto, Auburn University, USA

Nadja Møbjerg, University of Copenhagen, Denmark

Marie Monniaux, University of Lille, France

Bibiana Montoya, Autonomous University of Tlaxcala, Mexico

Christopher Morrow, Harvard University, USA

Raegan Mozal, Butler University, USA

Amrita Mukherjee, University of Cambridge, UK

Mansi Mungee, Azim Premji University, India

Perumal Nagarajan, National Institute of Immunology, India

Takeshi Nakamura, Tokyo University of Science, Japan

Patrick Narbonne, University of Quebec in Trois-Rivières, Canada

Shumaila Naz, University of Wah, Pakistan

Francesca Nazio, University of Rome Tor Vergata, Italy

K. A. I. Nekaris, Oxford Brookes University, UK

Joana Neves, King's College London, UK

James Nieh, University of California, San Diego, USA

Mikko Nikinmaa, University of Turku, Finland

Zehra Nizami, PartitionBio Ltd, UK

Hétor Nolasco-Soria, Northwest Biological Research Center (CIBNOR), Mexico

Tommy Norin, Technical University of Denmark, Denmark

Leila Nuri, Oakland University, USA

Tristan O'Harrow, University of Pennsylvania, USA

Jordana Oliveira, University of Ottawa, Canada

Cintia Oliveira Carvalho, Federal University of Pará, Brazil

Brent Opell, Virginia Polytechnic Institute and State University, USA

Xilma Ortiz-Gonzalez, University of Pennsylvania, USA

Pierre Osteil, University of Clermont Auvergne, France

Indira Paddibhatla, Johns Hopkins University School of Medicine, USA

Johnny Padulo, University of Milan, Italy

Oné Pagán, West Chester University, USA

Isabel Palacios, Queen Mary University of London, UK

Matthew Pamenter, University of Ottawa, Canada

Tyler Paul, State University of New York Upstate Medical University, USA

Jun Wei Pek, Temasek Life Sciences Laboratory Ltd, Singapore

Jarrod Petersen, Brown University, USA

Daisy Pineda-Suazo, National Autonomous University of Mexico, Mexico

Dilek Pirim, Bursa Uludağ University, Turkey

Berenika Plusa, The University of Manchester, UK

Konrad Pomianowski, Polish Academy of Sciences, Poland

Sean Powers, University of Richmond, USA

Prasanta Jana, Birsa Agricultural University, India

David Pruyne, State University of New York Upstate Medical University, USA

Alexa Rabeling, University of Cape Town, South Africa

Glenn Radice, The Warren Alpert Medical School of Brown University, USA

Sini Rahuman, Bahrain Polytechnic, Bahrain

Hamed Rajabi, London South Bank University, UK

Hamid Rajabiesterabadi, Islamic Azad University, Islamic Republic of Iran

Nirupama Ramadas, University of North Carolina at Chapel Hill, USA

Arjun Ramakrishnan, Indian Institute of Technology Kanpur, India

Stephan Reber, Lund University, Sweden

Deran Reddy, University of the Witwatersrand, South Africa

Gregory Redpath, University of New South Wales, Australia

Jörg Renkawitz, Ludwig Maximilian University of Munich, Germany

Jessica Ridilla, State University of New York Upstate Medical University, USA

Thomas Roberts, Brown University, USA

Paul Robinson, University of Oxford, UK

Samuel Robinson, Institute for Wetland and Waterfowl Research, Canada

Dulce Robles Martinez, California State University San Marcos, USA

Michael Romero, Mayo Clinic College of Medicine and Science, USA

Wan Rong, Baise University, China

Sharath Rongali, Missouri State University-West Plains, USA

Chris Rongo, Rutgers University, USA

Leone Rossetti, King's College London, UK

Arpita Roy, Sharda University, India

Conglin Ruan, University of Minnesota, USA

Kyle Rugg, Louisiana Tech University, USA

Peter Rugg-Gunn, Babraham Institute, UK

Bill Ryerson, Cornell University College of Veterinary Medicine, USA

Umair Safdar, University of Peshawar, Pakistan

Andreas Sagner, University of Erlangen-Nuremberg, Germany

Pablo Sambucetti, University of Buenos Aires, Argentina

Pierre Santucci, LISM Aix-Marseille University, France

Sajedeh Sarlak, University of Tehran, Islamic Republic of Iran

Noriaki Sasai, Nara Institute of Science and Technology, Japan

John Sayer, Newcastle University, UK

Miriam Schmidts, University Hospital Freiburg, Germany

Martin Schnittler, University of Greifswald, Germany

Violaine See, University of Liverpool, UK

Martina Semenzato, University of Padua, Italy

Lavinia Sheets, Washington University in St Louis, USA

Xiao Li Shen, Zunyi Medical University, China

Hiroko Shigemi, University of Fukui, Japan

Satoko Shinjo, University of Padua, Italy

Susan Shorter, University of Greenwich, UK

Sandra Shumway, University of Connecticut, USA

Anette Siggervåg, University of Bergen, Norway

Filipa Simões, University of Oxford, UK

Kenneth Smallwood, Independent Researcher, USA

Ashley Smiley, University of California, Berkeley, USA

Euncheol Son, University of Ulsan College of Medicine, Republic of Korea

Yuanquan Song, University of Pennsylvania, USA

Federica Spani, Campus Bio-Medico University of Rome, Italy

Sara Stahley, Penn State College of Medicine, USA

Flavie Strappazzon, Santa Lucia Foundation, Italy

Tohru Sugawara, Yokohama City University, Japan

Fei Sun, Duke University, USA

Rajivgandhi Sundaram, Johns Hopkins University School of Medicine, USA

Per Sunnerhagen, University of Gothenburg, Sweden

Diego Sustaita, California State University San Marcos, USA

Yim Tong Savio Szeto, Tung Wah College, Hong Kong

Ho Man Tang, Chinese University of Hong Kong, Hong Kong

Loïc Teulier, Claude Bernard University Lyon 1, France

Gary Thomas, University of Pittsburgh, USA

Joseph Thompson, Franklin & Marshall College, USA

Nathan Thompson, New York Institute of Technology, USA

Bret Tobalske, University of Montana, USA

Dhanendra Tomar, Wake Forest University School of Medicine, USA

Ewa Tomaszewska, University of Life Sciences in Lublin, Poland

Ashish Toshniwal, The University of Utah, USA

George Toworfe, Regent University College of Science and Technology, UK

Dan Tracey, Indiana University Bloomington, USA

Hanh Tran, Vietnam National University HCMC, Vietnam

Miltos Tsiantis, Max Planck Institute for Plant Breeding Research, Germany

Jeffrey Tuhtan, Tallinn University of Technology, Estonia

M.V. Ushakov, Voronezh State University, Russia

Somaye Vaissi, Razi University, Islamic Republic of Iran

Franco Valdez Ovallez, National University of San Juan, Argentina

Merel Van Gorp, University of Antwerp, Belgium

Yegor Vassetzky, Institute Gustave Roussy, France

Deepika Vasudevan, University of Pittsburgh, USA

Katrina Velle, University of Massachusetts Amherst, USA

Nancy Walworth, Rutgers University, USA

Dehua Wang, Shandong University, China

Na Wang, The Third Hospital of Hebei Medical University, China

Shuoshuo Wang, Beth Israel Deaconess Medical Center, USA

Pablo Wappner, Leloir Institute Foundation, Argentina

Lesley Weaver, Indiana University Bloomington, USA

Barbara Webb, The University of Edinburgh, UK

Stefanie Weidtkamp-Peters, Heinrich Heine University Düsseldorf, Germany

Bregje Wertheim, University of Groningen, Netherlands

Benedikt Westermann, Institute of Cell Biology and Neurobiology, Germany

Brooke Whitelaw, University of Otago, New Zealand

Alexander Whitworth, University of Cambridge, UK

Jessie Williamson, Cornell University, USA

Thad Wilson, University of Kentucky College of Medicine, USA

Nicholas Wu, Western Sydney University, Australia

Suhui Wu, Henan University of Chinese Medicine, China

Ye Xiong, Lund University, Sweden

Kang Nian Yap, Norwegian University of Science and Technology, Norway

Amelia Yeap, Khoo Teck Puat Hospital, Singapore

Jaeho Yoon, National Cancer Institute, USA

Yuki Yoshida, National Agriculture and Food Research Organization, Japan

Taishi Yoshii, Okayama University, Japan

Aya Yoshimura, Fujita Health University, Japan

Fengwei Yu, Temasek Life Sciences Laboratory (TLL), Singapore

Guangcheng Yue, Henan University of Science and Technology, China

Catherine Zainathan, University of Malaysia, Terengganu, Malaysia

Georgia Zarkada, University of Connecticut, USA

Luca Zinzula, Max Planck Institute of Biochemistry, Germany
